# The Relationship of Tumor Microbiome and Oral Bacteria and Intestinal Dysbiosis in Canine Mammary Tumor

**DOI:** 10.3390/ijms231810928

**Published:** 2022-09-18

**Authors:** Hui-Hua Zheng, Chong-Tao Du, Chao Yu, Xin-Yue Tang, Rong-Lei Huang, Yu-Zhu Zhang, Wei Gao, Guang-Hong Xie

**Affiliations:** 1 College of Veterinary Medicine, Jilin University, Changchun 130062, China; 2 Department of Laboratory Animals, College of Animal Sciences, Jilin University, Changchun 130062, China

**Keywords:** canine mammary tumors, microbiome, intratumoral microbiome, oral microbiome, gut microbiome

## Abstract

Canine mammary tumor (CMT) is the most common tumor in dogs, with 50% of malignant cases, and lacks an effective therapeutic schedule, hence its early diagnosis is of great importance to achieve a good prognosis. Microbiota is believed to play important roles in systemic diseases, including cancers. In this study, 91 tumors, 21 oral and fecal samples in total were collected from dogs with CMTs, and 31 oral and 21 fecal samples from healthy dogs were collected as control. The intratumoral, oral and gut bacterial community of dogs with CMTs and healthy dogs was profiled by 16S rRNA high-throughput sequencing and bioinformatic methods. The predominant intratumoral microbes were *Ralstonia*, *Allorhizobium-Neorhizobium-Pararhizobium-Rhizobium*, *Pseudomonas*, *unidentified_Chloroplast* and *Bacteroides* at the genus level. In addition, our findings demonstrated striking changes in the composition of the oral and gut bacterium community in the dogs suffered from CMTs compared to the healthy dogs, with a significant increase of *Bacteroides* which also was the significant microbial biomarker in the oral and gut bacterium community. It showed that the *Bacteroides* was shared in the intratumoral, oral and intestinal bacterial microbiomes, confirming that microbiota might travel from the mouth to the intestine and finally to the distant mammary tumor tissue. This study provides a new microbiological idea for the treatment of canine mammary tumors, and also provides a theoretical basis for the study of human breast cancer.

## 1. Introduction

In recent years, more and more domestic pets are raised as the spiritual sustenance of humans, particularly dogs. The morbidities of various diseases have increased with the rise of the dog population and the prolongation of the life span, especially tumors [1]. As the most common type of tumors, canine mammary tumor (CMT) usually occurs in 5 years old or older bitches, with approximately 50% of malignant cases [2,3]. Due to the common features of mammary gland tumors between dogs and humans, CMT was an excellent model for human breast cancer studies [4]. Breast cancer is widely known as a multifactorial malignancy, and the risk factors for development include host factors (non-modifiable) and external factors (modifiable), which can influence the host microbiome [5]. Over 16% of cancer morbidity in the whole world has been caused by infectious microbes, which contained viruses, bacteria, and parasites [6,7]. The vast populations of microbial species live in and on the human body, and play an important role in the host body [5,8,9]. Increasing evidence suggested that microbe-host interactions have the potential influence on the development of breast cancer [10,11,12].

In the past decade, some studies reported that the microbiome could either directly (within the tumor) or indirectly (within the gut or oral) increase the chances of developing cancers, for example, liver, cervical, colorectal, pancreatic, oral, and breast [11,12,13,14]. As the direct factor that influences the development of tumors, intratumor bacteria was first discovered 100 years ago, but its characterization was not confirmed by the 16S rRNA sequencing until 2020 [11]. In Nejman’s study, seven cancer types had different rich and diverse microbiomes, with a higher bacterial load and richness in breast cancer which enriched the top three bacteria *Proteobacteria*, *Firmicutes* and *Actinobacteria* at phyla [11]. On the indirect factor, gut microbes might be capable of quickly migrating in the bloodstream and then to distant tissues, further affecting the development of tumors [14,15,16]. Furthermore, another indirect factor, the oral dysbacteriosis were also associated with the progression of both local and remote tumors, and could impact the risk of cancers, including oral, breast, lung and hematological cancers [13,17,18,19,20]. Interestingly, there were some reports that tumors had similar bacterial profiles to the intestine in pancreatic cancer, and the alteration in the oral, gastrointestinal, as well as pancreatic microbiota, can lead to the development of pancreatic cancer [14,21]. As human companion animals, dogs live in the same environment as human, and has been considered an excellent model for human breast cancer study. However, so far, its characterization of intratumor, gut and oral microbiome remains unclear, and the relationship between the three is also blank in the international arena.

Therefore, based on this background, we hypothesized that the risk of CMTs might be related to the composition and functionality of the mammary tumor, gut or oral microbiota. In this study, we aimed to explore the characterization of the intratumor, gut and oral microbiome in dogs with canine mammary tumors using 16S rRNA high-throughput sequencing, and to reveal whether intratumor microbiota can move from the gastrointestinal tract or oral cavity to the mammary gland on dogs.

## 2. Results

### 2.1. Clinical Characteristics of Dogs

**Figure 1:**
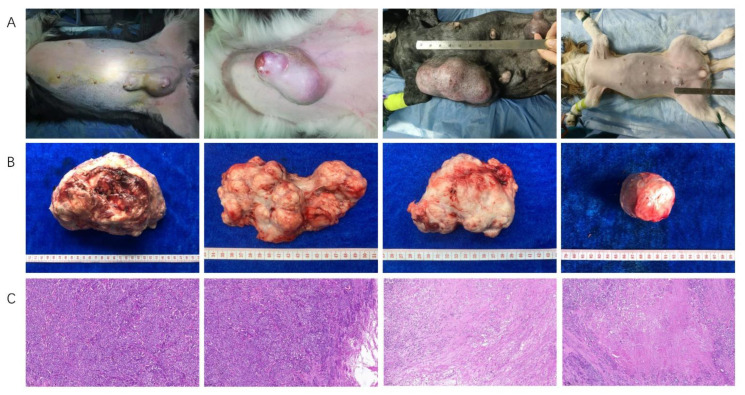
The feature of dogs with canine mammary tumors. (**A**) The clinical features of canine mammary tumors in dogs, and single and multiple tumor(s) were observed in a dog; (**B**) The sizes and shapes of canine mammary tumors after an excisional mastectomy, and the different sizes, unclear boundaries and irregular shapes were observed in vitro; (**C**) Histopathological diagnosis of canine mammary tumors in dogs, which were the cell morphology under low magnification (100×).

To characterize the CMT-associated tissue, gut and oral microbiome, the tumor, feces and oral swabs of lateral oral gingival mucosa from dogs with CMTs and its control samples including the normal tissue adjacent to tumors from diseased dogs, feces and oral swabs from healthy dogs were collected and analyzed during September 2020 and July 2021. A total of 147 dogs were collected in this study, which included 95 dogs with CMTs and 52 healthy dogs. Among them, we collected 91, 21 and 21 tumors (Figure 1), feces and oral swabs from 95 dogs with CMT, respectively, with 23 of the normal tumor-adjacent tissue, 21 feces and 31 oral swabs from 52 healthy dogs corresponding control samples. The age, gender, breed, somatotype and country were uniformity both two groups (Table S1).

### 2.2. Intratumoral Microbial Composition and Diversity

**Figure 2:**
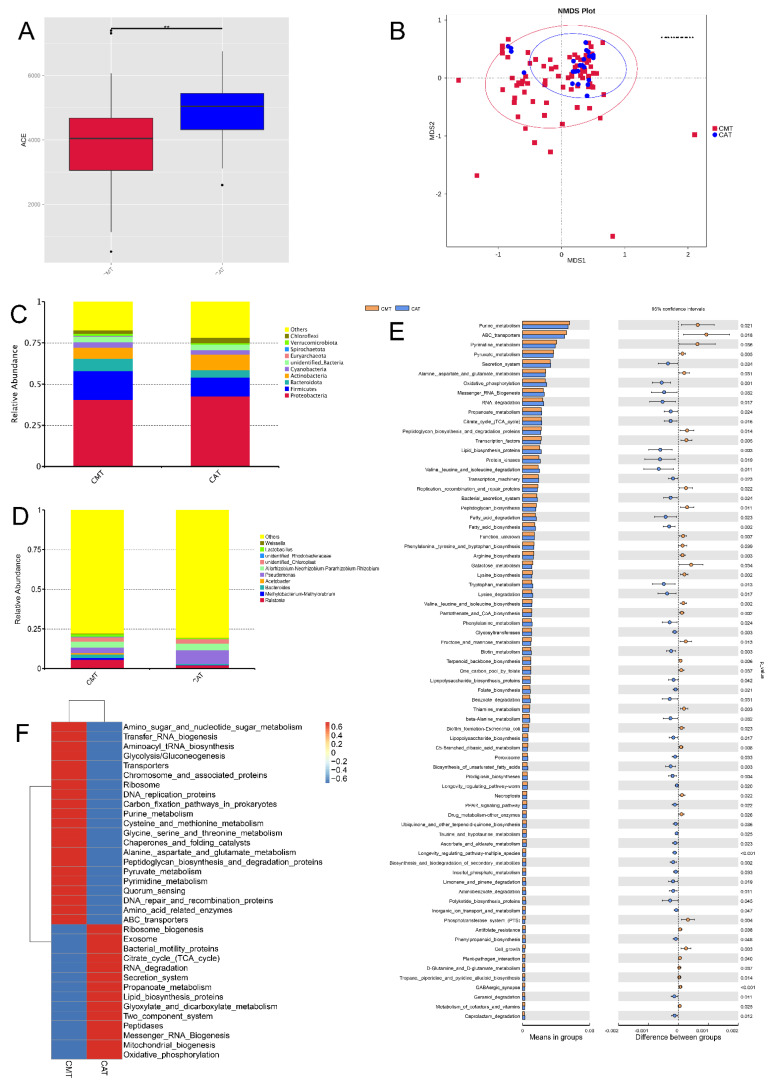
Comparisons of microbiota diversity between canine mammary tumor tissues (CMT) and canine tumor-adjacent tissues (CAT). (**A**) Alpha diversity (ACE index) of microbial communities between both CMT and CAT groups: a decrease in with-sample bacterial richness was observed in CMT group. (**B**) Beta diversity (analysis of similarity) of microbial community structures between both CMT and CAT groups: there was no obvious difference between both of them (*p* > 0.05). (**C**,**D**) The distribution of top 10 phyla (**C**) and top 10 genera (**D**) detected in canine mammary tumor tissues; (**E**) KEGG pathways which displayed significant difference with CMT and CAT groups: 32 pathways were upregulated and 43 pathways were downregulated; (**F**) The value of log10 relative fold between both CMT and CAT groups for 35 pathways: the changes were found. Statistically significant differences (*p* < 0.05) are indicated by **.

To analyze the bacterial composition and differences among canine mammary tumors (CMTs) and canine tumor-adjacent tissues (CAT), we applied high-throughput sequencing to detect the V4 region of the bacterial 16S rRNA gene. As a result, 78,586 raw sequence reads with an average length of 253 bps were produced from all samples. In total, 5200 OTUs were obtained from the sequencing results, which included 121phyla, 271 classes, 585 orders, 876 families, 1883 genera and 1464 species. The results of the ACE index (alpha diversity) showed that a decrease in within-sample bacterial richness was observed in those intratumor (Figure 2A), which was in agreement with the result of other richness index chao1 (Figure S1A). What’s more, analyses of sample-to-sample differences in microbial community structures (Beta diversity) showed that there was no obvious difference between CMT and CAT groups (analysis of similarity, *p* > 0.05) (Figure 2B). To characterize the intratumoral microbial composition, we conducted a taxonomic analysis and detected a predominance of phylum *Proteobacteria* (40.64%; i.e.,40.64% of the overall reads sequenced), *Firmicutes* (17.56%), *Bacteroidota* (7.35%) and *Actinobacteria* (6.77%) (Figure 2C), and the *Firmicutes*, *Myxococcota* and *Campylobacterota* of CMT group were significant different that of CAT group (Figure S1B). At the genus level, the most prevalent ones were *Ralstonia* (5.75%), *Allorhizobium-Neorhizobium-Pararhizobium-Rhizobium* (3.77%), *Pseudomonas* (3.44%), *unidentified_Chloroplast* (2.78%) and *Bacteroides* (2.21%) (Figure 2D), which there was a significant difference of the *Ralstonia* between groups (Figure S1C).

### 2.3. Functional Prediction of Intratumoral Microbiota Related to the Development of CMT

To characterize the changes in biological functions that may be involved in the CMT and CAT groups, the Tax4Fun toolkit was adopted to predict KEGG pathways. As a result, 304 KEGG pathways were predicted, and 73 were shown notably different between the CMT and CAT groups based on the number of sequences that are correlated with the pathways (*p* < 0.05), of which 32 pathways were upregulated and 43 pathways were downregulated (Figure 2E), and Figure 2F portrayed the value of log10 relative fold change between groups for 35 pathways.

With a focus on pathways relevant to microbial metabolism, we found that pathway modules associated with nucleotide metabolism, enzyme families, xenobiotics biodegradation and metabolism, metabolism of other amino acids and metabolism of terpenoids and polyketides were differentially enriched between the CMT and CAT groups (*p* < 0.05). Particularly, there were six of the 73 pathways involved in the glycan biosynthesis and metabolism, which were peptidoglycan biosynthesis and degradation proteins, peptidoglycan biosynthesis, glycosyltransferases, lipopolysaccharide biosynthesis proteins, lipopolysaccharide biosynthesis, and glycosylphosphatidylinositol (GPI) anchored proteins. Overall, our results present here reveal CMT-associated alterations in the composition and functionality of intratumoral microbiota.

### 2.4. Oral Microbial Composition and Diversity between the Canine Mammary Tumor and Healthy Dogs

**Figure 3:**
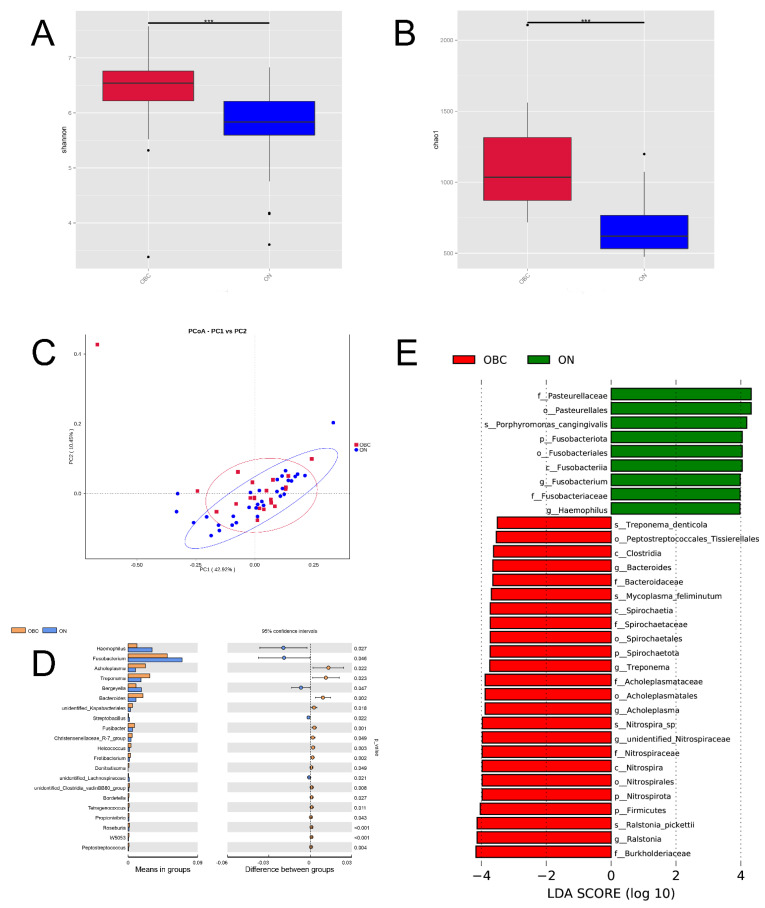
Oral microbial composition and diversity in dogs with canine mammary tumors (OBC group) and healthy dogs (ONN group). (**A**,**B**) Alpha diversity (Shannon and Chao1 index) of microbial communities between both CMT and CAT groups: two mean values of the OBC group were both higher than that of the ONN group; (**C**) Beta diversity (the weighted UniFrac analysis) of microbial community structures between both OBC and ONN groups: it depicted the distance relationship between both two groups; (**D**) The oral microorganisms which had significantly different abundance in the OBC and ONN groups at genus level; (**E**) The LDA Effect Size (LEfSe) of OBC and ONN groups: it exhibited the remarkable bacteria of oral microbiota that characterizes the differences between both two groups. Statistically significant differences (*p* < 0.01) are indicated by ***.

To analyze the oral bacterial composition and differences between the dogs suffering from CMTs (OBC group) and healthy dogs (ONN group), we applied high-throughput sequencing to detect the V4 region of the bacterial 16S rRNA gene. As a result, 4,363,630 raw sequence reads with an average length of 253 bp were produced from all the oral swabs. In total, 2286 OTUs (operational taxonomic units) were obtained from the sequencing results, which include 58 phyla, 142 classes, 307 orders, 466 families, 777 genera, and 536 species. The Shannon and Chao1 indexes explicitly showed that the differences in oral bacterial community composition existed between the OBC and ONN groups, and two mean values of the OBC group (6.331, SD = 0.8617; 1112.022, SD = 341.3) were both higher than that of the ONN group (5.729, SD = 0.724; 669.679, SD = 189.7) (Figure 3A,B). Moreover, the mean values of Simpson and ACE indexes in the OBC group were both higher than that of the ONN group (Figure S2A,B). As shown in Figure 3C, the weighted UniFrac analysis depicted the distance relationship between samples of the OBC and ONN groups. Anosim analysis showed that the classification of the two groups of samples yielded a *p* value of 0.03 and an R value of 0.09 (Figure S2C), which indicated that the among-group difference was greater than the intragroup difference.

To further reveal the significance of grouping, we performed an analysis of NMDS, and the results exhibited that the stress value of OBC and ONN groups was 0.127 (less than 0.2) (Figure S2D). To sum up, the two groups in this study have statistically significant differences in the oral bacterial community structure, and the grouping of OBC and ONN is the most explanatory. To characterize the oral microbial composition, we conducted a taxonomic analysis and detected a predominance of phylum *Proteobacteria* (32.41%), *Bacteroidota* (31.31%) and *Firmicutes* (10.26%), with a less common presence of *Spirochaetota* (2.89%) on the OBC group (Figure S2E). At the genus level, the most prevalent ones were *Porphyromonas* (18.84%), *Fusobacterium* (5.07%), *Moraxella* (4.84%), *Ralstonia* (3.61%) and *Conchiformibius* (2.88%), with a slight presence of *Bacteroides* (1.94%) on the OBC group (Figure S2F). Since disturbance in the relative abundance of oral microbiota was observed at the genus level (Figure 3D), we sought for correlation of specific oral microorganisms at the genus level with CMT. Taking stringent criteria (higher than 0.1% in abundance and presence in more than 90% of swabs), six genera with significant differences in the levels between the OBC and ONN groups were identified as the core CMT-associated microbiota (Figure 3D). We found that the OBC group harbored higher levels of *Acholeplasma*, *Treponema* and *Bacteroides* than that of the ONN group, with the decreases of *Haemophilus*, *Fusobacterium* and *Bergeyella*. Additionally, LDA Effect Size (LEfSe) was performed to further uncover the remarkable species of oral microbiota that characterize the differences between the OBC and ONN groups (Figure 3E). When the log10 (LDA score) was greater than 3.5, the dominant bacteria in the OBC group were *Bacteroides*, *Spirochaeta*, *Firmicutes* and *Nitrospirota* at the phylum level, and the dominant bacteria in the ONN group were *Fusobacteriota* and *Pasteurellales*.

### 2.5. Gut Microbial Composition and Diversity between the Canine Mammary Tumor and Healthy Dogs

**Figure 4:**
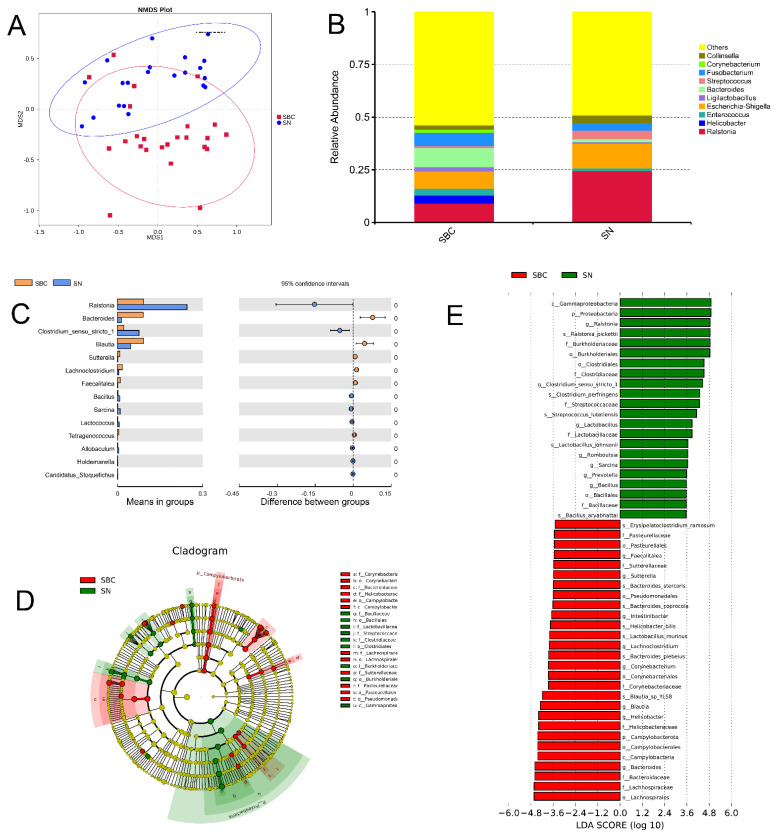
Gut microbial composition and diversity in dogs with canine mammary tumors (SBC group) and healthy dogs (SNN group). (**A**). Beta diversity (the analysis of similarity) of microbial community structures between both SBC and SNN groups: the gut microbiome of SBC group clustered separately from that of the control ONN group; (**B**) The distribution of the top 10 genera detected in fecal samples of dogs with canine mammary tumors; (**C**) The gut microorganisms which had significantly different abundance in SBC and SNN groups at genus level; (**D**) The cladogram of the gut microorganism of SBC and SNN groups; (**E**) The LDA Effect Size (LEfSe) of SBC and SNN groups: it displayed the remarkable species of gut microbiota between both two groups, and its log 10 (LDA score) was greater than 3.5.

To analyze the gut bacterial composition and differences between the dogs suffering from CMTs (SBC group) and healthy dogs (SNN group), we applied high-throughput sequencing to detect the V4 region of the bacterial 16S rRNA gene. As a result, 3,797,952 raw sequence reads with an average length of 253 bp were produced from all the oral swabs. In total, 2447 OTUs (operational taxonomic units) were obtained from the sequencing results, which include 63 phyla, 148 classes, 316 orders, 465 families, 900 genera, and 554 species. The alpha diversity showed that there were no significant differences in gut bacterial community composition existed between SBC and SNN groups (*p* > 0.05). Moreover, analyses of sample-to-sample differences in microbial community structures (beta diversity) showed that the gut microbiome of the SBC group clustered separately from that of the control ONN group (analysis of similarity, *p* = 0.001) (Figure 4A). To characterize the gut microbial composition of the SBC group, we conducted a taxonomic analysis and detected a predominance of phylum *Firmicutes* (47.65%), *Proteobacteria* (21.96%), *Bacteroidota* (11.02%) and *Fusobacteriota* (7.94%) (Figure S3A), and Proteobacteria and *Bacteroidota* showed a significant difference between groups (Figure S3B). At the genus level, the most prevalent ones were *Bacteroides* (9.08%), *Ralstonia* (9.19%), *Escherichia-Shigella* (8.34%) and *Fusobacterium* (6.33%) (Figure 4B), which Bacteroides and Ralstonia had a significant difference between groups (Figure 4C). Additionally, to further uncover the remarkable species of gut microbiota that characterize the differences between the groups, we predicted the biomarkers for the SBC and SNN group by considering both statistical significance and biological consistency with linear discriminant analysis of effect size (Figure 4D). When the log 10 (LDA score) was greater than 3.5, the dominant bacteria in the SBC group were *Bacteroides*, *Blautia* and *Helicobacter* at the genus level, and the dominant bacteria in the SNN group were *Ralstonia* and *Clostridium* (Figure 4E).

### 2.6. The Relationship between Intratumoral Bacterial Taxa and Oral and Gut Microbiome

**Figure 5:**
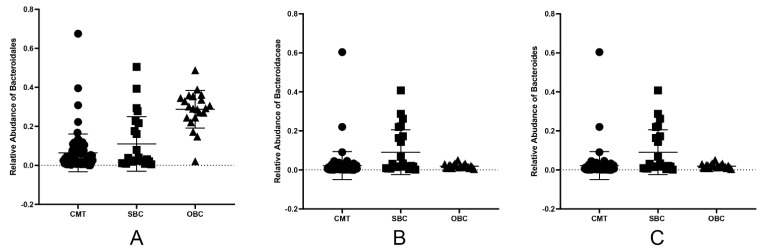
The relative abundance of common microbiota in tumors, gut and oral samples of dogs with CMTs. The *Bacteroidales*, *Bacteroidaceae* and *Bacteroides* were detected in canine mammary tumors, and were found in both disordered oral and gut microbiota. (**A**) The relative abundance of *Bacteroidales* in tumors (CMT group), gut (SBC group) and oral (OBC group) samples of dogs with CMTs; (**B**,**C**) The relative abundance of *Bacteroidaceae* (**B**) and *Bacteroides* (**C**) in CMT, SBC and OBC groups. Black circles, black squares and black triangle represent tumor, feces and oral swabs samples, respectively.

Since the intratumoral microbiota and the disturbance in the relative abundance of oral and gut microbiota were observed (Figure 2C, Figure 3D and Figure 4C), we sought the correlation of specific intratumoral microorganisms with disordered oral and gut microbiota. As a result, *Ralstonia*, *Bacteroides* and *Lactobacillus* were the predominances of genus among in tumor, gut and oral microbiome. *Bacteroides* in the tumor, gut and oral microbiome of diseased dogs were higher than these of healthy dogs. That says, the *Bacteroidales*, *Bacteroidaceae* and *Bacteroides* were detected in the tumor, and were also observed in the disordered oral and gut microbiota (Figure 5), which were identified as the core CMT-associated microbiota (Figure 3E and Figure 4E). It proved that microbiota might travel from the mouth to the intestine and finally to the distant mammary tissue. In addition, two oral and one gut microbiota with significant differences in the genus level between tumor and control groups were identified as the core CMT-associated microbiota, and these microbiotas were also detected in the tumors.

### 2.7. Isolation and Identification of the Tumoral Bacteria

The bacterial isolates were examined based on culture characteristics, and it was observed that most of the isolates formed off-white pinhead colonies typical of *Propionibacterium* and *Streptococcus* spp. The identification of isolated *Propionibacterium* and *Streptococcus* spp. from canine mammary tumor samples was determined using the 16S rRNA gene by PCR analysis. The sequences were aligned to the query sequences of the GenBank 16S rRNA sequences database, three isolated *Propionibacterium acnes* and one *Streptococcus anginosus* were obtained, and their genus was also found in the gut and oral bacterium by the 16S rRNA sequencing.

## 3. Discussion

Canine mammary tumor was one of the most common types of tumors, with approximately 50% of malignant cases [2,3]. CMT is highly invasive with a poor prognosis and lacks effective therapeutic drugs. In the past decade, some studies reported that the microbiome could either directly (within the tumor) or indirectly (within the gut or oral) increase cancer risk. Therefore, analysis of the characterization of the microbiome is important for the prevention at an early stage of CMT is of crucial importance. As stated above, the oral cavity is characterized by the highest microbiome density and possesses the largest number of species in the body, and oral bacteria have been proven associated with many cancers, as the gastrointestinal tumors, oral cancer and pancreatic cancer [13,22,23]. The gut bacteria can influence the activation of the immune system promoting cancer-associated inflammation and ultimately affecting tumor responses to therapies, such as the gut microbiota as a host factor can mediate tumor responses to chemotherapy and immunotherapy in patients with melanoma and lung cancers [24,25,26,27]. Additionally, the intratumoral microbiota factors may determine tumor behavior and patient outcomes [11,28]. The characterization of the intratumor microbiome was first analyzed in 2020, and the predominant bacteria in breast tumors were *Proteobacteria*, *Firmicutes* and *Actinobacteria* at phyla [11]. Hence, we firstly found associations between canine mammary tumors and the microbial community composition. In this study, we analyzed the bacterial composition of CMT by the 16S rRNA high-throughput sequencing technology. The predominant intratumor microbes were *Proteobacteria*, *Firmicutes*, *Bacteroidota* and *Actinobacteria* at the phylum level (Figure 1C), which was similar to these of human breast cancer [11,29]. At the genus level, the most prevalent ones were *Ralstonia*, *Allorhizobium-Neorhizobium-Pararhizobium-Rhizobium*, *Pseudomonas*, *unidentified_Chloroplast* and *Bacteroides* (Figure 1D), and among them, *Pseudomonas* and *Ralstonia* were also dominant genera in human breast cancer [29].

Due to many host parameters being shown to modify the oral and gut microbiome such as gender, breed and age, careful control for analyzing the microbiome between tumor and healthy dogs was necessary. Most of the bacterial communities found in the tumoral milieu are present commonly in the gut microbiome [28,30]. Herein, we analyzed the composition of the gut bacterial community, the predominant gut microbes of the SBC group were *Proteobacteria*, *Firmicutes*, *Bacteroidota* and *Actinobacteria* at the phylum level (Figure 1C), and the most prevalent ones were *Ralstonia*, *Allorhizobium-Neorhizobium-Pararhizobium-Rhizobium*, *Pseudomonas*, *unidentified_Chloroplast* and *Bacteroides* at the genus level (Figure 1D). Discovering biomarkers has proven to be the most important and successful way to translate molecular and genomic research output into clinical applications. Our efforts systematically shed light on the differences in gut bacterial community composition between the SBC and SNN groups. At the phylum level, the SBC group tended to have a higher percentage of *Bacteroidetes* and lower percentages of Proteobacteria (*p* < 0.05). At the genus level, we observed significant increases in the abundances of *Bacteroides* and *Blautia* in the SBC group compared with the SNN group, with a significant decrease of *Ralstonia* (*p* < 0.05). In addition, the LEfSe analysis further revealed *Bacteroides* were characteristically enriched in the SBC groups (Figure 3D). Simultaneously, *Bacteroides* was also detected in the tumor, suggesting that potentially bacterial translocation from the gut to the mammary might be occurring.

Many studies indicated that members of the oral microbiota are involved in intestinal dysbiosis, indirectly affecting the composition of the intestinal microbiota via dissemination into the gut [31]. It was demonstrated that the majority (54%) of the patient-enriched, taxonomically assigned members of intestinal microbiota originated from the oral cavity by using metagenomics and gene catalogs [32]. Subsequently, several studies have recently examined and validated that some microbiota were shared between oral and feces between patients with cancers and healthy humans [23,31]. To verify the presence of common microbiota both in the oral and fecal samples of dogs with CMTs, we first characterized the oral microbial composition, and the results showed that the top three microbiota at the phylum were *Proteobacteria*, *Bacteroidota* and *Firmicutes*, and the most prevalent ones were *Porphyromona*, *Fusobacterium*, *Moraxella*, *Ralstonia* and *Conchiformibius*, with a slight presence of *Bacteroides* at the genus level. It could be seen that there was some common microbiota both in OBC and SBC groups, such as *Proteobacteria*, *Bacteroidota* and *Firmicutes* of phyla and *Ralstonia* and *Bacteroides* of the genus, which was corresponding with the previous study [23,28]. Furthermore, we explored the species diversity of oral bacterial communities between OBC and ONN groups, and the performance of the two groups on the Simpson uncovered that the oral bacteria species diversity of the OBC group was higher than that of the ONN group, which was also supported by the other species diversity index Shannon (Figure 1A). The differences are perhaps related indirectly to the biological functions of the canine mammary glands because of the imbalance in the body’s microbiome. Furthermore, our efforts systematically shed light on the differences in oral bacterial community composition among the OBC and ONN groups. Compared to the ONN group, the OBC group had higher percentages of *Spirochaetota* and *Desulfobacrerota* and a lower percentage of *Fusobacteriota* at the phylum level, and had higher abundances of *Acholeplasma*, *Treponema* and *Bacteroides* and lower abundances of *Heamophilus*, *Fusobacterium* and *Bergeyella* at the genus level (*p* < 0.05). Subsequently, LEfSe analysis further revealed that the significant the microbial biomarker included *Bacteroides*, *Treponema*, *Acholeplasma*, *unidentified_Nitrospiraceae* and *Ralstonia* at the genus level on the OBC group. Therefore, we conclude that the high abundance of *Treponema* and *Bacteroides*, may stand out as potential specific risk factors for CMTs.

So far, there was no related research on whether the CMT contained bacteria and its source. This study first reported the bacterial composition of CMT, which contained a high abundance of *Bacteroides*, suggesting that *Bacteroides* might be related to the occurrence and development of CMT. The result was similar to the previous study, but the latter difference was that the *Bacteroides fragilis* was observed in cancerous breasts, and induced the growth and metastatic progression [33]. What’s more, our findings demonstrated striking changes in the composition of the oral and gut bacterium community in the dogs suffered from CMT compared to the healthy dogs, with a significant increase of *Bacteroides* which also was the significant microbial biomarker on the oral and gut bacterium community. It can be seen that *Bacteroides* in the oral and gut microbiota were present in the tumor, indicating that microbiota might travel from the mouth to the intestine and finally to the distant mammary tissue. This result might explain why the risk of breast cancer in patients with periodontitis associated with oral bacterial disorders increased [19]. In veterinary medicine, periodontal diseases are very common in dogs, with 44–64% of dogs being affected by the disease, and oral microbiota plays a prominent role in periodontal disease pathogenesis [34]. It further implied a potentially increased risk of CMTs in dogs with oral microbiota dysbiosis.

Our study determined the characteristics of intratumoral microbiota by the 16S rRNA high-throughput sequencing, and confirmed the intestinal and oral dysbacteriosis of diseased dogs with CMTs. Additionally, the presence of common bacteria among three sites of dogs indicated that microbiota might travel from the oral cavity to the intestine and finally to the distant mammary tumor tissue. It was beneficial to develop a new microbiological method for the prevention and treatment of canine mammary tumors, and played a considerable role in other tumors of dogs and human breast cancer studies.

## 4. Materials and Methods

### 4.1. Sample Collection

Tumoral, fecal and oral samples from the dogs suspected of suffering CMTs and normal tissue adjacent to the suspected tumor, feces and oral swabs from the healthy dogs were collected from the animal hospital in Changchun province, China, between September 2020 and June 2021. The age, breed, gender, somatotype, spayed status and region of healthy dogs were in agreement with that of diseased dogs suffering CMTs (Table S1). The procedures of samples were as follows: the internal feces were immediately collected into the sterile tubes after the defecation of dogs, and the oral swabs mainly scraped the lateral gingival mucosa of dogs. After the excisional mastectomy on dogs suffering from tumors, fresh tissues of tumors and normal tissue adjacent to tumors were immediately transferred to germ free 50 mL conical tubes with sterile DMEM culture medium, and processed in the clean and sterile cell culture dishes with autoclaved dissection tools under ultra clean cabinet. Treated samples were rapidly put into the sterile tubes, and then stored at −80 °C until use.

### 4.2. Histopathology

For histological studies, the CMTs were fixed in 10% neutral buffered formalin. After fixation the material was washed in saline phosphate buffer (PBS), followed by dehydration in a series of ethanol in increasing concentrations (from 70 to 100%), followed by diaphanization in xylol and paraffin inclusion. The paraffin blocks were submitted to microtomy (Leica, RM2165, Wetzlar, Germany) and the cuts of 5 μm made were adhered on histological slides and left in an oven at 60 °C for 12 h. After that, the slices of the glands fragments on the slides underwent a routine of tissue staining techniques using Hematoxylin and Eosin, Masson’s Trichrome and Picrosirius Red. Next, the analysis and photos were made through normal and polarized light on a Leica BX80 Camera Zaiss HRC microscope.

The canine mammary gland tissues were interpreted by an expert pathologist and those with CMTs defined by histopathology were considered CMT cases.

### 4.3. DNA Extraction and Bacterial 16S rRNA Sequencing

Genomic DNA was extracted from each sample using DNA Miniprep Kit (Omega, Norcross, GA, USA) according to the manufacturer’s instructions, and then were used as the templates for PCR amplification of the V4 region of the 16S rRNA gene. PCR was conducted in a 30 μL volume mixture including in 15 μL Phusion^®^ Gigh-Fidelity PCR Mastere Mix GC Budder 2× (New England Biolabs company, UK), 10 μL the extracted gDNA template (1 ng/μL), 3 μL H_2_O and 2 μL of 2 μM 16S rRNA V4 (515F/806R) barcoded primer mix which was consisted of equimolar forward (515F: 5′-GTGCCAGCMGCCGCGGTAA-3′) and reverse (806R: 5′-GGACTACHVGGGTWTCTAAT-3′) primer pairs [35]. The cycling protocol was an initial denaturation at 98 °C for 1 min, followed by 30 cycles at 98 °C for 10 s, 50 °C for 30 s, and 72 °C for 30 s, with a final step of 72 °C for 5 min. DNA from the phosphate buffered saline was used as a negative control. PCR amplicons were detected by electrophoresis in a 2% agarose gel containing ethidium bromide, and were purified using the GeneJET Gel Extraction Kit (Thermo Scientific, Waltham, MA, USA) following to manufacturer’s recommendations. Subsequently, the sequencing libraries were constructed by the TruSeq DNA PCR-Free Library Preparation Kit (Illumina, San Diego, CA, USA) according to manufacturers’ recommendations and index codes were added. Purified libraries were evaluated on the Qubit@2.0 Fluorometer (Thermo Scientific) and Agilent Bioanalyzer 2100 system, and employed for cluster generation with 250 bp paired-end reads and sequencing in the NovaSeq 6000 platform (Illumina) [36].

### 4.4. Data Processing

Due to the presence of dirty data, the merging, quality filtering and removal of chimeric reads were performed in raw data which was obtained after sequencing using the QIIME 2 pipeline [37] to acquire the effective data, and then those data were clustered into operational taxonomic units (OTU) at the 97% sequence homogeneity. According to the OTUs clustering results, the representative sequences of each OTU are annotated to obtain the corresponding species information and abundance distribution based on species. Meanwhile, to analyze the microbial community diversity in the sample [38], the indexes of alpha diversity analysis including community diversity (Shannon and Simpson diversity indices), evenness (Shannon equitability index) and richness (Chao1) were statistics. For evaluating the beta diversity, the weighted and unweighted UniFrac parameters [39] were calculated by using the QIIME pipeline. Non-metric dimensional scaling was conducted using the weighted correlation network analysis, stat and ggplot2 packages in R software by transforming a distance matrix of weighted or unweighted UniFrac parameters among samples into a new set of orthogonal axes.

Functional composition of metagenomes was predicted from 16S rRNA data by the PICRUSt software [40], the pipeline of which is composed of two workflows, gene content prediction and metagenome prediction. A table of gene copy numbers for each gene family in each sequenced bacterial and archaeal genome based on the IMG database [41] and a phylogenetic tree from the Greengenes database [42] was precomputed for gene content prediction. Subsequently, metagenome prediction was performed by multiplying the vector of gene counts for each OTU by the abundance of that OTU in each sample, and summed across all OTUs.

### 4.5. Isolations of Tumoral Bacteria

For isolating tumoral bacteria, tumor tissues were placed in 75% ethanol solution for 3 min to disinfect the surface, and then vortexed multiple times in the sterile PBS buffer to encourage the removal of any bacteria on the tissue surface. Subsequently, the epidermis of tumors was removed using a new blade and sterile cell culture to obtain the tissues within the tumorous. Finally, these acquired internal tumor tissues were divided into two sections. One treated tumor was incubated in solid medium plates with 5% sterile defibrinated sheep blood, including brain heart infusion (BHI) and columbia agar base medium, for 3–5d at a 37 °C incubator under anaerobic conditions [43]. A single colony that had different forms was picked with a sterilized wire loop and re-streaked on corresponding blood agar plates for purification. The process was repeated until the purified single colony was obtained. Another part of the tumor was sheared and incubated in a corresponding broth medium with 5% fetal bovine serum at 37 °C for 3–5d under the same condition, and then two broth mediums were assessed for turbidity. The turbid broth medium caused by microorganism growth was inoculated in solid medium plates for 3–5d at 37 °C under anaerobic conditions. Different bacterial strains were isolated and purified by the same method. The anaerobic condition for inoculating plates was provided by AnaeroPack pouch bags (Mitsubishi Gas Chemical, Tokyo, Japan).

### 4.6. Identification of the Isolated Bacteria

The isolated tumoral bacteria were identified based on their characterization of the colonies, morphological observation and 16S rRNA gene sequencing. The purified bacteria were serially diluted with sterile saline solution, and coated on blood agar plates followed by incubation at 37 °C for 3d under the anaerobic condition to observe the characterization of the colonies. The morphology of the isolated strains was observed by Gram staining. Subsequently, the turbid broth medium was sent to Comate Bioscience Co., Ltd (Changchun, Jilin province, China). for sequencing, and the obtained sequences were aligned to the GenBank database using NCBI BLAST.

### 4.7. Statistical Analysis

Shannon, Simpson, ACE and Chao1 indexes between canine mammary tumors and normal tissues adjacent to suspected tumors were compared by using Student’s *t*-test. The discrimination in community composition between diseased and normal groups was determined by analysis of similarities of UniFrac parameters using 999 permutations in each test. Significant changes in the phylum and genus relative abundance of the microbiome were analyzed by Student’s t-test and adjusted by Benjamini-Hochberg correction. Statistically significant biomarkers at the OTU level were identified by the linear discriminant analysis of effect size analysis [44]. It was considered a significant difference in which the *p* value was less than 0.05.

## 5. Conclusions

In summary, we first systematically described the characteristics of intratumoral bacterium composition in the canine mammary tumor, and analyzed the oral and gut bacterial microbiome composition and differences between the dogs with CMTs and healthy dogs. The result showed that the *Bacteroides* was shared in the intratumoral, oral and intestinal bacterial microbiomes, confirming that microbiota might travel from the mouth to the intestine and finally to the distant mammary tumor tissue. This study provides a new microbiological idea for the treatment of canine mammary tumors, and also provides a theoretical basis for the study of human breast cancer.

## Data Availability

All data generated or analyzed during this study are available in National Center for Biotechnology Information BioProject database with accession numbers PRJNA866706 and PRJNA866710.

## Supplementary Materials

The following supporting information can be downloaded at: https://www.mdpi.com/article/10.3390/ijms231810928/s1.
