# Supplementary material for: The Relationship of Tumor Microbiome and Oral Bacteria and Intestinal Dysbiosis in Canine Mammary Tumor
Source: Int J Mol Sci. 2022 Sep 18;23(18):10928. doi: 10.3390/ijms231810928 (PMC9503607; doi:10.3390/ijms231810928)
Supplement: Supplementary file 1 [file ijms-23-10928-s001.zip › ijms-1906480-supplementary.pdf]

Figure S1 Comparisons of microbiota diversity between canine mammary tumor tissues (CMT) and canine tumor-adjacent tissues (CAT). A. Alpha diversity (Chao1 index) of microbial communities between both CMT group and CAT group; B and C. The intratumoral microbiota which had significantly different abundance in CMT and CAT groups at phylum (B) and genus (C) levels.

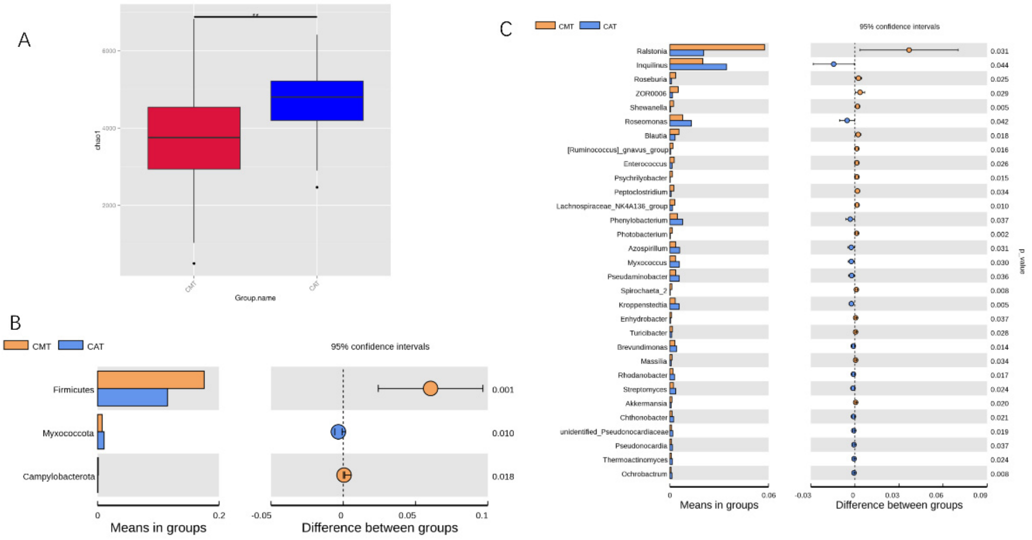

Figure S2 Oral microbial composition and diversity in diseased dogs with canine mammary tumors (OBC group) and healthy dogs (ONN group). A and B. Alpha diversity (Simpson and ACE index) of oral microbial communities in OBC and ONN groups: they had a significant difference between both two groups; C. Beta diversity (Anosim analysis) of microbial community structures between both OBC and ONN groups; D. The Non-Metric Multi-Dimensional Scaling (NMDS) analysis of two groups: it exhibited that the stress value of OBC and ONN groups was 0.127 (less than 0.2); E and F. The distribution of top 10 phyla (E) and top 10 genera (F) detected in OBC and ONN groups.

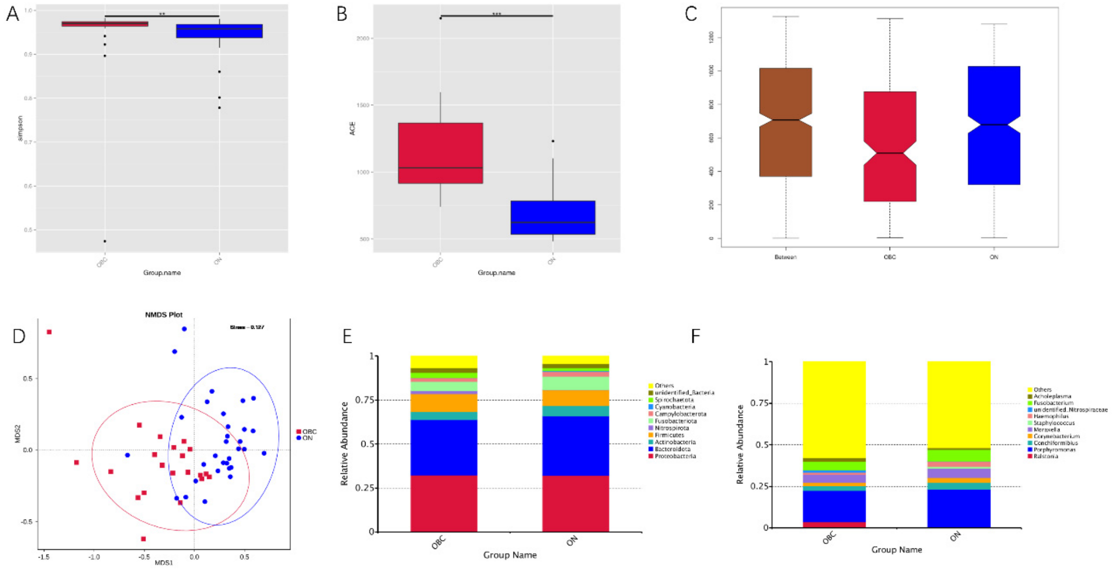

Figure S3 The gut microbial composition and diversity in diseased dogs with canine mammary tumors (SBC group) and healthy dogs (SNN group). A. The distribution of top 10 phyla detected in SBC and SNN groups; B. The gut microorganisms which had significantly different abundance in SBC and SNN groups at genus level.

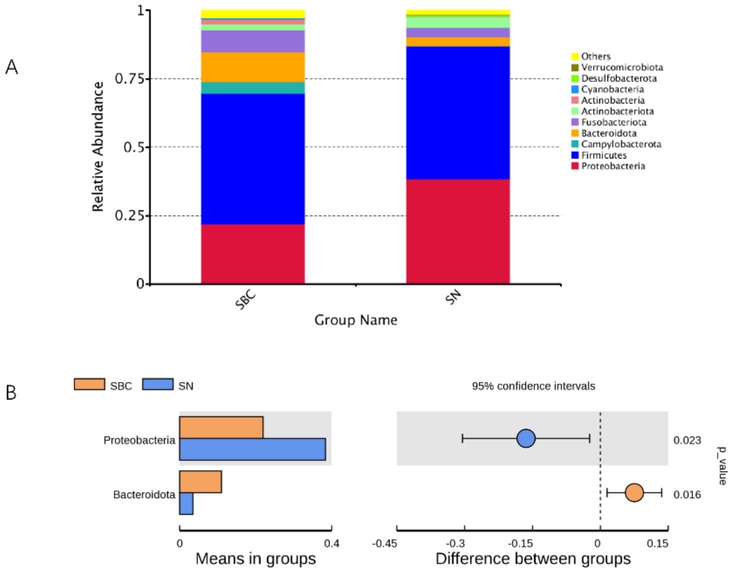

Table S1 The clinical information of healthy dogs and diseased dogs with canine mammary tumors

Health status of dogs	Tumor tissue	Tumor-adjacent tissue	Oral swab	Faeces	Breed	Somatotype	Age (years)	Gender	Spayed status	Pathologic diagnosis

Cannine mammary tumor	DB001	—	—	SBC001~3	Samoyed	Medium	10	Female	No	Malignant
Cannine mammary tumor	DB002	—	—	—	Poodle	Small	16	Female	No	Malignant
Cannine mammary tumor	DB003	—	—	—	Poodle	Small	16	Female	No	Malignant
Cannine mammary tumor	—	NBC003	—	—	Teddydog	Small	12	Female	No	Malignant
Cannine mammary tumor	DB004	—	OBC004	—	Cocker	Small	11	Female	Yes	Malignant
Cannine mammary tumor	DB005	—	—	—	Bobbie	Small	14	Female	No	Benign
Cannine mammary tumor	DB006	—	—	—	Bulldog	Medium	4	Female	Yes	Benign
Cannine mammary tumor	DB007	—	—	SBC007	Italian Greyhound	Small	12	Female	No	Malignant
Cannine mammary tumor	DB008	—	OBC008	SBC008	Mixed breed	Medium	12	Female	Yes	Malignant
Cannine mammary tumor	DB010	—	OBC009	SBC009	Pekingese	Small	12	Female	No	Benign
Cannine mammary tumor	DB011	—	OBC010	SBC010	Teddydog	Small	13	Female	No	Benign
Cannine mammary tumor	DB012	—	OBC011	SBC011	Old English Sheepdog	Large	14	Female	No	Malignant
Cannine mammary tumor	DB013	—	OBC012	SBC012	Tibetan Mastiff	Large	10	Female	No	Malignant
Cannine mammary tumor	—	—	OBC013	SBC013	Teddydog	Small	8	Female	No	Benign
Cannine mammary tumor	DB014	DBH014	OBC014	SBC014	Golden retriever	Large	12	Female	No	Malignant
Cannine mammary tumor	DB015	—	OBC015	SBC015	Teddydog	Small	9	Female	Yes	Benign
Cannine mammary tumor	—	—	OBC016	—	Mixed breed	Medium	9	Female	No	Benign
Cannine mammary tumor	—	DNB017	OBC017	SBC017	Teddydog	Small	8	Female	No	Benign
Cannine mammary tumor	DB016	DBH016	OBC018	SBC018	Schnauzer	Small	12	Female	Yes	Benign
Cannine mammary tumor	DB017	—	OBC019	SBC019	Teddydog	Small	5	Female	No	Malignant
Cannine mammary tumor	DB018	DNB218	—	—	Teddydog	Small	9	Female	No	Benign
Cannine mammary tumor	DB019	—	OBC021	SBC021	Mixed breed	Medium	13	Female	No	Benign
Cannine mammary tumor	DB020	—	OBC022	SBC022	Samoyed	Medium	13	Female	Yes	Malignant
Cannine mammary tumor	DB021	—	—	—	Samoyed	Medium	13	Female	Yes	Malignant
Cannine mammary tumor	DB022	—	OBC023	SBC023	Border Collie	Medium	8	Female	No	Malignant
Cannine mammary tumor	DB023	—	—	—	Border Collie	Medium	8	Female	No	Malignant
Cannine mammary tumor	DB024	—	—	—	Border Collie	Medium	8	Female	No	Malignant
Cannine mammary tumor	DB025	—	OBC024	SBC024	Mixed breed	Small	14	Female	No	Malignant
Cannine mammary tumor	DB026	DBH026	—	—	Teddydog	Small	7	Male	No	Malignant

Cannine mammary tumor	DB027	DBH027	—	—	Mixed breed	Medium	13	Female	No	Benign
Cannine mammary tumor	DB028	—	—	—	Teddydog	Small	8	Female	No	Malignant
Cannine mammary tumor	DB029	—	—	—	Samoyed	Medium	7	Female	No	Benign
Cannine mammary tumor	DB030	—	—	—	Samoyed	Medium	8	Female	No	Malignant
Cannine mammary tumor	DB031	—	—	—	Teddydog	Small	9	Female	No	Malignant
Cannine mammary tumor	DB032	—	—	—	Teddydog	Small	12	Female	No	Malignant
Cannine mammary tumor	DB033	—	—	—	Samoyed	Medium	11	Female	No	Malignant
Cannine mammary tumor	DB034	—	—	—	Papillon	Small	10	Female	No	Benign
Cannine mammary tumor	DB035	—	—	—	Teddydog	Small	7	Female	No	Malignant
Cannine mammary tumor	DB036	—	—	—	Samoyed	Medium	9	Female	No	Malignant
Cannine mammary tumor	DBF037	DNB037	—	—	Teddydog	Small	11	Female	Yes	Benign
Cannine mammary tumor	DBF038	—	—	—	Pomeranian	Small	11	Female	No	Benign
Cannine mammary tumor	DBF039	—	—	—	Pomeranian	Small	11	Female	No	Benign
Cannine mammary tumor	DBF040	DNB040	OBC032	SBC032	Schnauzer	Small	3	Female	No	Benign
Cannine mammary tumor	DBF041	—	—	—	Schnauzer	Small	3	Female	No	Benign
Cannine mammary tumor	DBF042	—	—	—	Pomeranian	Small	8	Female	No	Benign
Cannine mammary tumor	DBC043	DNB043	OBC034	SBC034	Teddydog	Small	7	Female	No	Malignant
Cannine mammary tumor	DBC044	—	—	—	Mixed breed	Medium	13	Female	Yes	Malignant
Cannine mammary tumor	DBC045	NBC045	—	—	Teddydog	Small	12	Female	No	Malignant
Cannine mammary tumor	DBC046	—	—	—	Teddydog	Small	8	Female	No	Malignant
Cannine mammary tumor	DBC047	—	—	—	Teddydog	Small	12	Female	No	Malignant
Cannine mammary tumor	DBC048	—	—	—	Teddydog	Small	12	Female	No	Malignant
Cannine mammary tumor	DBC049	NBC049	OBC037	SBC037	Border Collie	Medium	9	Female	Yes	Malignant
Cannine mammary tumor	DBC050	NBC050	—	—	Pomeranian	Small	11	Female	Yes	Malignant
Cannine mammary tumor	DBC051	NBC051	—	—	Border Collie	Medium	9	Female	Yes	Malignant
Cannine mammary tumor	DBC052	—	—	—	Teddydog	Small	10	Female	No	Malignant
Cannine mammary tumor	DBC053	—	—	—	Teddydog	Small	9	Female	Yes	Malignant
Cannine mammary tumor	DBC054	—	—	—	Teddydog	Small	10	Female	Yes	Malignant
Cannine mammary tumor	DBC055	—	—	—	Teddydog	Small	8	Female	No	Malignant

Cannine mammary tumor	DBC056	—	—	—	Border Collie	Medium	9	Female	No	Maligna nt
Cannine mammary tumor	DBC057	—	—	—	Pomeranian	Small	11	Female	No	Maligna nt
Cannine mammary tumor	DBC058	—	—	—	Border Collie	Medium	9	Female	No	Maligna nt
Cannine mammary tumor	DBC059	NBC059	—	—	Border Collie	Medium	9	Female	No	Maligna nt
Cannine mammary tumor	DBC060	NBC060	—	—	Bichon Frise	Small	10	Female	No	Maligna nt
Cannine mammary tumor	DBC061	NBC061	—	—	Border Collie	Medium	9	Female	No	Maligna nt
Cannine mammary tumor	DBC062	—	—	—	Border Collie	Medium	9	Female	No	Maligna nt
Cannine mammary tumor	DBC063	—	—	—	Bichon Frise	Small	12	Female	No	Maligna nt
Cannine mammary tumor	DBC064	—	—	—	Border Collie	Medium	9	Female	No	Maligna nt
Cannine mammary tumor	DBC065	—	—	—	Border Collie	Medium	9	Female	Yes	Maligna nt
Cannine mammary tumor	DBC066	—	—	—	Bichon Frise	Small	7	Female	No	Maligna nt
Cannine mammary tumor	DBC067	—	—	—	Border Collie	Medium	9	Female	Yes	Maligna nt
Cannine mammary tumor	DBC068	—	—	—	Border Collie	Medium	11	Female	Yes	Maligna nt
Cannine mammary tumor	DBC069	—	—	—	Mixed breed	Medium	4	Female	No	Benign
Cannine mammary tumor	DBC070	—	—	—	Border Collie	Medium	9	Female	No	Maligna nt
Cannine mammary tumor	DBC071	—	—	—	Border Collie	Medium	14	Female	No	Maligna nt
Cannine mammary tumor	DBC072	—	—	—	Poodle	Small	6	Female	No	Maligna nt
Cannine mammary tumor	DBC073	—	—	—	Border Collie	Medium	9	Female	No	Maligna nt
Cannine mammary tumor	DBC074	—	—	—	Welsh corgi pembroke	Small	9	Female	No	Maligna nt
Cannine mammary tumor	DBC075	NBC075	—	—	Border Collie	Medium	9	Female	Yes	Maligna nt
Cannine mammary tumor	DBC076	NBC076	—	—	Border Collie	Medium	9	Female	Yes	Maligna nt
Cannine mammary tumor	DBC077	NBC077	—	—	Golden retriever	Large	5	Female	No	Maligna nt
Cannine mammary tumor	DBC078	—	—	—	Border Collie	Medium	9	Female	Yes	Maligna nt
Cannine mammary tumor	DBC079	—	—	—	Shiba Inu	Medium	10	Female	Yes	Maligna nt
Cannine mammary tumor	DBC080	—	—	—	Border Collie	Medium	9	Female	Yes	Maligna nt
Cannine mammary tumor	DBF084	—	—	—	Alaskan Malamute	Large	5	Female	No	Benign
Cannine mammary tumor	DBF085	—	—	—	Alaskan Malamute	Large	8	Female	No	Benign
Cannine mammary tumor	DBF086	—	—	—	Alaskan Malamute	Large	5	Female	No	Benign
Cannine mammary tumor	DB091	—	—	—	Poodle	Small	7	Female	No	Maligna nt

Cannine mammary tumor	DB092	—	—	—	Poodle	Small	6	Female	No	Malignant
Cannine mammary tumor	DB093	—	OBC041	SBC041	Teddydog	Small	10	Female	No	Malignant
Cannine mammary tumor	DB094	NBC094	—	—	Teddydog	Small	10	Female	No	Malignant
Cannine mammary tumor	DBF095	NBC095	—	—	Teddydog	Small	10	Female	No	Benign
Cannine mammary tumor	DB096	NBC096	—	—	Teddydog	Small	12	Female	No	Malignant
Cannine mammary tumor	DB097	—	—	—	Teddydog	Small	13	Female	No	Malignant
Cannine mammary tumor	DB098	—	—	—	Teddydog	Small	10	Female	No	Malignant
Cannine mammary tumor	DBF099	—	—	—	Poodle	Small	9	Female	No	Benign
Heath	—	—	—	SN053	Papillon	Small	2	Female	No	—
Heath	—	—	—	SN054	Teddydog	Small	7	Female	No	—
Heath	—	—	—	SN055	Teddydog	Small	8	Female	No	—
Heath	—	—	—	SN056	Teddydog	Small	12	Female	No	—
Heath	—	—	—	SN061	Shiba Inu	Medium	9	Female	No	—
Heath	—	—	—	SN062	Golden retriever	Large	6	Female	No	—
Heath	—	—	—	SN063	Bichon Frise	Small	10	Female	No	—
Heath	—	—	—	SN067	Golden retriever	Large	5	Female	No	—
Heath	—	—	—	SN070	Golden retriever	Large	7	Female	No	—
Heath	—	—	—	SN071	Bichon Frise	Small	11	Female	No	—
Heath	—	—	—	SN072	Bichon Frise	Small	7	Female	No	—
Heath	—	—	—	SN077	Chihuahua	Small	12	Female	No	—
Heath	—	—	—	SN080	Pomeranian	Small	10	Female	No	—
Heath	—	—	—	SN084	Bichon Frise	Small	9	Female	No	—
Heath	—	—	—	SN091	Shiba Inu	Medium	11	Female	No	—
Heath	—	—	—	SN092	French bulldog	Medium	13	Female	No	—
Heath	—	—	—	SN093	Mixed breed	Medium	9	Female	No	—
Heath	—	—	—	SN101	Border Collie	Medium	9	Female	No	—
Heath	—	—	—	SN105	Teddydog	Small	14	Female	No	—
Heath	—	—	—	SN107	Teddydog	Small	16	Female	No	—
Heath	—	—	—	SN115	Schnauzer	Small	10	Female	No	—
Heath	—	—	ON006	—	Teddydog	Small	5	Female	No	—
Heath	—	—	ON011	—	Mixed breed	Medium	12	Female	No	—
Heath	—	—	ON014	—	Pomeranian	Small	12	Female	No	—
Heath	—	—	ON017	—	Teddydog	Small	13	Female	No	—
Heath	—	—	ON022	—	Golden retriever	Large	3	Female	No	—
Heath	—	—	ON025	—	Bichon Frise	Small	8	Female	No	—
Heath	—	—	ON028	—	Chow Chow	Large	9	Female	No	—
Heath	—	—	ON036	—	Bichon Frise	Small	5	Female	No	—
Heath	—	—	ON052	—	Bichon Frise	Small	11	Female	No	—
Heath	—	—	ON058	—	Bichon Frise	Small	10	Female	No	—
Heath	—	—	ON059	—	Bichon Frise	Small	9	Female	No	—
Heath	—	—	ON063	—	Bichon Frise	Small	8	Female	No	—
Heath	—	—	ON074	—	Cocker	Small	5	Female	No	—
Heath	—	—	ONN001	—	Bichon Frise	Small	10	Female	No	—
Heath	—	—	ONN003	—	Teddydog	Small	9	Female	No	—
Heath	—	—	ONN004	—	Bichon Frise	Small	10	Female	No	—
Heath	—	—	ONN005	—	Shih Tzu	Small	11	Female	No	—
Heath	—	—	ONN007	—	Welsh corgi pembroke	Small	12	Female	No	—
Heath	—	—	ONN010	—	Shih Tzu	Small	5	Female	No	—
Heath	—	—	ONN011	—	Teddydog	Small	9	Female	No	—

Heath	—	—	ONN013	—	Teddydog	Small	12	Female	No	—
Heath	—	—	ONN014	—	Mixed breed	Medium	13	Female	Yes	—
Heath	—	—	ONN016	—	Teddydog	Small	9	Female	Yes	—
Heath	—	—	ONN017	—	Mixed breed	Medium	8	Female	Yes	—
Heath	—	—	ONN023	—	Mixed breed	Medium	9	Female	Yes	—
Heath	—	—	ONN025	—	French bulldog	Medium	10	Female	Yes	—
Heath	—	—	ONN026	—	French bulldog	Medium	8	Female	Yes	—
Heath	—	—	ONN027	—	Mixed breed	Medium	11	Female	No	—
Heath	—	—	ONN028	—	Mixed breed	Medium	10	Female	Yes	—
Heath	—	—	ONN029	—	Teddydog	Small	11	Female	No	—
Heath	—	—	ONN030	—	Shih Tzu	Small	10	Female	Yes	—
